# Decreased intracellular zinc in human tumorigenic prostate epithelial cells: a possible role in prostate cancer progression

**DOI:** 10.1186/1475-2867-6-10

**Published:** 2006-03-31

**Authors:** Liping Huang, Catherine P Kirschke, Yunfan Zhang

**Affiliations:** 1 Western Human Nutrition Research Center/Agriculture Research Service/United States Department of Agriculture, Davis, California, USA; 2 Department of Nutrition, University of California at Davis, Davis, California, USA; 3 Rowe Program in Genetics, University of California at Davis, Davis, California, USA; 4 Department of Anatomy and Cell biology, Wayne State University, Detroit, Michigan, USA

## Abstract

**Background:**

Zinc plays important roles in maintaining normal function of the prostate and in development of prostate malignancy. It has been demonstrated that prostate malignant epithelial cells contain much less cellular zinc than the surrounding normal epithelial cells. However, the pathway(s) which leads to lower zinc accumulation in malignant prostate epithelial cells is poorly understood. In this study, the zinc homeostatic features of two human prostate epithelial cell lines (non-tumorigenic, RWPE1, and tumorigenic, RWPE2) were investigated. Effects of over-expression of ZIP1 in RWPE2 on cell proliferation and apoptosis were also studied.

**Results:**

RWPE2 accumulated less intracellular zinc than RWPE1 due to the decreased zinc uptake activity. The mRNA expression of ZIP1 and ZIP3 in RWPE1 and RWPE2 was comparable. However, the protein expression of ZIP1 in RWPE2 was lower than that in RWPE1. ZIP3 was detected in a lysosomal compartment of RWPE2 while no ZIP3 was detected in the same compartment of RWPE1. Over-expression of ZIP1 in RWPE2 resulted in an elevation of intracellular zinc concentration and suppression of cell growth of RWPE2 due to the increased apoptosis.

**Conclusion:**

These findings suggest that tumorigenic prostate epithelial cells accumulated less intracellular zinc than non-tumorigenic prostate epithelial cells. The reduction in capacity for accumulation of intracellular zinc in tumorigenic prostate epithelial cells may be caused by the decrease in the ZIP1 protein expression and the intracellular redistribution of ZIP3 in RWPE2. RWPE1 and RWPE2 are excellent cellular models to study the association of intracellular zinc levels with prostate cancer progression.

## Background

It is well known that normal human prostate glands accumulate almost 10-fold higher zinc as compared to other soft tissues, such as liver and kidney [1,2]. Accumulation of cellular zinc and secretion of zinc into the prostatic fluid in prostate glands are essential functions of the prostate secretory epithelial cell [3]. Additionally, low zinc concentration in seminal plasma may affect the mobility of sperm which can result in infertility in men [4-6]. The glandular epithelial cells in the dorsolateral lobe of the prostate accumulate the highest levels of intracellular zinc [3,7]. This is also the area where most prostate cancers occur. This observation strongly suggests that zinc has an important role in prostate cancer development and/or progression. Studies have indicated that the intracellular zinc levels in malignant prostate epithelial cells are dramatically decreased [8,9]. This contrasts with benign prostatic hyperplasia in which the epithelial cells accumulate normal or higher levels of zinc [8,9]. The reduction of intracellular zinc concentrations in prostate epithelial cells may promote prostate cancer initiation and/or progression via cell cycle arrest, programmed cell death, necrosis, or oxidative stress [2,10-14]. Although zinc is an important factor in prostate biology and pathology, the exact roles of zinc and its homeostatic regulation in the normal and malignant prostate glands are not understood.

Recent advances in the study of molecules involved in intracellular zinc homeostasis, such as zinc transporters, have given us a hope to dissect the molecular mechanisms of how the high zinc levels are maintained in prostate epithelial cells under normal conditions and how the prostate epithelial cells become "zinc deficient" under malignant conditions. Two families of zinc-transporter proteins, ZnT (Zinc Transporter) and ZIP (ZRT1, IRT1-like protein) have been identified in mice and human [15,16]. The ZnT proteins, which are the members of the CDF family (cation diffusion facilitator), appear to function either by transporting zinc out of the cell or by sequestering zinc into intracellular compartments [16]. In contrast, the ZIP proteins appear to function in uptake of extracellular or in release of stored zinc into the cytoplasm [15]. At present, 24 zinc transporters (10 ZnT and 14 ZIP proteins) have been identified through genomic sequence analysis in mammals. Thirteen of them have been functionally characterized [17-28]. These studies have indicated that zinc transporters act in tissue, cell type, and organelle specific manner with some functional redundancy. These specialized zinc transporters maintain intracellular zinc concentrations in a narrow physiologic range.

The activities of zinc transporters are regulated by extracellular zinc concentrations via transcription, translation, and protein trafficking. For example, in zinc-replete conditions, the expression of ZnT1 mRNA and protein is up-regulated [29]. Meanwhile, ZIP1 and ZIP3 are rapidly internalized from the plasma membrane to intracellular compartments accompanied by the redistributions of the ZnT4 and ZnT6 proteins from their Golgi apparatus to the periphery of the cell [22,30]. These zinc-induced regulations via either decreasing zinc influx and/or increasing zinc efflux maintain the cellular zinc concentration at the level adequate for their physiologic targets while minimizing the toxicity of zinc excess.

Given the importance of zinc in the normal function of the prostate and in the development and/or progression of prostate cancer, we investigated the effects of extracellular zinc on zinc accumulation and on mRNA and protein expression of zinc transporters in two cultured prostate epithelial cell lines, RWPE1 and RWPE2. The RWPE1 cell line was immortalized with human papillomavirus 18 from histological normal prostate epithelial cells and the RWPE2 cell line was derived from RWPE1 by transforming the cells with Kirsten murine sarcoma virus (Ki-MuSV) [31]. RWPE1 and RWPE2 have retained normal epithelial cell morphology and both cell lines express cytokeratins 8 and 18, which are the markers for the prostate intermediate epithelial cell type with proliferative ability. Both cell lines are hormone sensitive and are positive for the androgen receptor expression. When cultured in the medium containing 5 nM mibolerone, both cell lines expressed PSA with higher levels detected in RWPE2. The RWPE2 cells can grow in soft agar *in vitro *and develop tumors in nude mice whereas the RWPE1 cells can not. So, RWPE1 and RWPE2 essentially share the same genotype differing only in their tumorigenic status [31]. In this study, we found that the capacity for zinc accumulation was decreased in RWPE2 because of the aberrant protein expression and cellular localization of zinc uptake proteins, ZIP1 and ZIP3. Over-expression of ZIP1 in RWPE2 increased intracellular zinc levels and suppressed cell growth through apoptosis.

## Results

### Decreased total cell-associated zinc level in tumorigenic prostate epithelial cells

**Figure 1:**
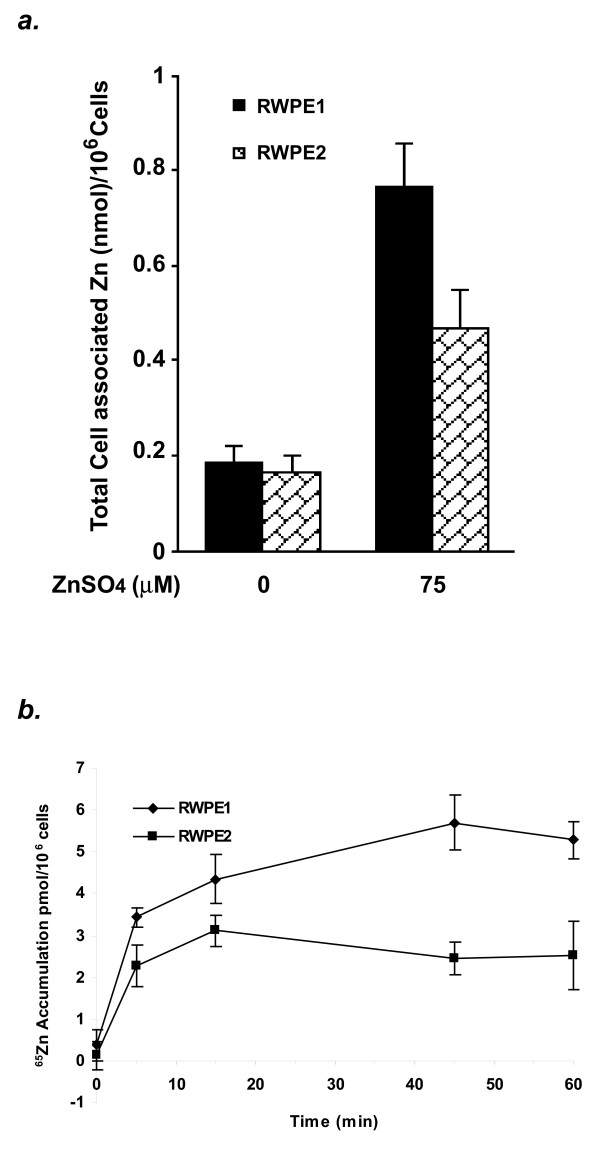
Cellular zinc accumulation in RWPE1 and RWPE2. *a.* Total cell-associated zinc contents. RWPE1 and RWPE2 were grown to ~50% confluence and treated with 0 or 75 μM ZnSO_4 _at 37°C for 48 hours. Cells were harvested and assayed for total cell-associated zinc contents by inductively coupled plasma-atomic emission spectrometry. Values are the means ± SE, n = 3 (three independent experiments). *b*. ^65^Zn accumulation. RWPE1 and RWPE2 were grown to ~70% confluence and harvested. Zinc accumulation was assayed with 0.05 μM ^65^ZnCl_2 _from 0 to 60 minutes. Values are the means ± SE, n = 3 (one representative experiment of three with similar results).

It has been demonstrated that prostate cancer tissues contain less cellular zinc compared to the surrounding non-cancerous tissues [8,9]. In order to elucidate the molecular basis underlying this clinical finding, we studied the zinc homeostatic features of two existing prostate epithelial cell lines, RWPE1, a non-tumorigenic prostate epithelial cell line, and RWPE2, its tumorigenic counterpart. We first examined the total cell-associated zinc levels in RWPE1 and RWPE2 by their ability to accumulate intracellular zinc while cultured in zinc repleted conditions. As shown in Fig. 1a, the total cell-associated zinc levels in RWPE1 and RWPE2 were not significantly different when both cell lines were cultured in the K-SFM supplemented with human recombinant epidermal growth factor and bovine pituitary extract, which contained about 0.7 μM zinc ions. However, the total cell-associated zinc level in RWPE2 was greatly reduced (39% reduction) compared to that in RWPE1 when cells were cultured in the medium containing 75 μM ZnSO_4 _at 37°C for 48 hours. The reduced ability to accumulate intracellular zinc in RWPE2 was further confirmed by a ^65^Zn accumulation analysis. The accumulation of ^65^Zn (0.05 μM) over a period of 60 minutes was performed in a zinc uptake buffer and the γ counts were measured. Consistent with the results obtained with total cell-associated zinc measurement, RWPE2 only accumulated 52% of ^65^Zn that was accumulated in RWPE1 (Fig. 1b). These results suggest that the ability to accumulate intracellular zinc is suppressed in RWPE2.

### Reduced ^65^Zn uptake in RWPE2

**Figure 2:**
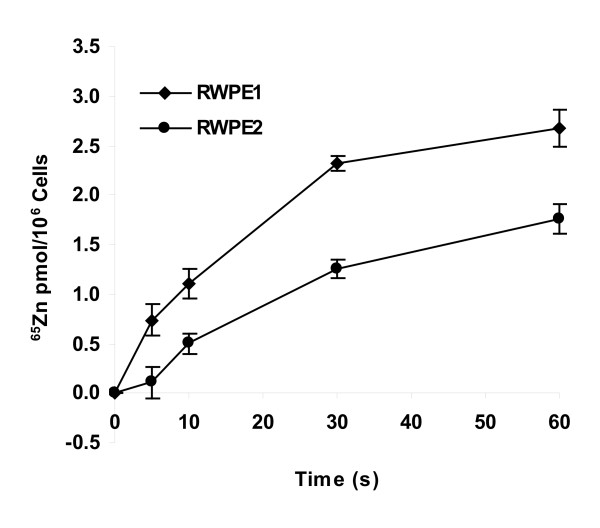
^65^Zn uptake in RWPE1 and RWPE2. RWPE1 and RWPE2 were grown to ~70% confluence and harvested. ^65^Zn uptake was assayed with 0.05 μM ^65^ZnCl_2 _from 0 to 60 seconds. Values are the means ± SE, n = 3 (one representative experiment of three with similar results).

We hypothesized that the zinc uptake system may be disrupted in RWPE2 which led to a reduction of intracellular zinc accumulation. Therefore, we examined the zinc uptake activities in RWPE1 and RWPE2 by determining the initial rates of ^65^Zn uptake. As shown in Fig. 2, RWPE1 accumulated zinc at an initial rate of 2.7 pmol/min/10^6 ^cells, whereas in RWPE2, that rate was decreased to 1.8 pmol/min/10^6^, a 33% reduction in zinc uptake activity. Taken together, these results strongly suggest that the decreased intracellular zinc accumulation in RWPE2 may be due to the incompetence of zinc uptake system of these cells.

### Expression of zinc transporters in RWPE1 and RWPE2

**Figure 3:**
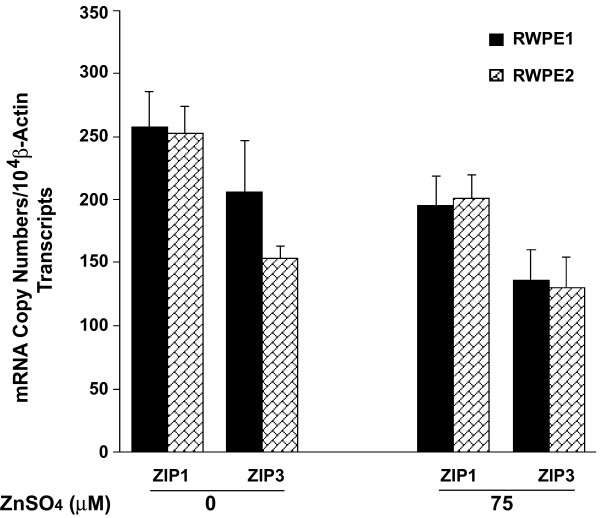
mRNA expression of zinc uptake transporters in RWPE1 and RWPE2. RWPE1 and RWPE2 were grown to ~50% confluence and treated with 0 or 75 μM ZnSO_4 _at 37°C for 48 hours. Cells were harvested and total RNA was isolated. Gene expression of ZIP1 and ZIP3 were measured by quantitative RT-PCR with TaqMan probes and normalized to the β-actin expression. The gene expression levels are presented as copy numbers/10^4 ^β-actin transcripts. Values are the means ± SE, n = 6 (two independent experiments).

To determine the molecular basis for the incompetence of the zinc uptake system in RWPE2, we first examined the mRNA expression profiles of zinc uptake transporters in RWPE1 and RWPE2. Total RNA was purified from RWPE1 and RWPE2 treated with either 0 or 75 μM ZnSO_4 _for 48 hours. The mRNA expression levels of four known zinc uptake proteins, ZIP1-4, were determined using a real-time quantitative RT-PCR analysis with TaqMan probes specific to these genes (Applied Biosystems). Although the abundance of the ZIP3 transcripts was about 20% and 40% less than that of ZIP1 in RWPE1 and RWPE2, respectively, the mRNA expression levels of ZIP1 and ZIP3 were similar between RWPE1 and RWPE2 (*p *> 0.05) (Fig. 3). In addition, both ZIP1 and ZIP3 mRNA expression was not affected by zinc treatment (*p *> 0.05, Fig. 3). Taken together, these results suggest that the discrepancy in zinc accumulation between RWPE1 and RWPE2 does not result from the transcriptional activities of the *ZIP1 *and *ZIP3 *genes. Using RT-PCR with TaqMan probes, the ZIP2 mRNA was barely detectable and no ZIP4 mRNA was detected (data not shown) in RWPE1 and RWPE2.

### Intracellular localization of ZIP1 and ZIP3 in RWPE1 and RWPE2

**Figure 4:**
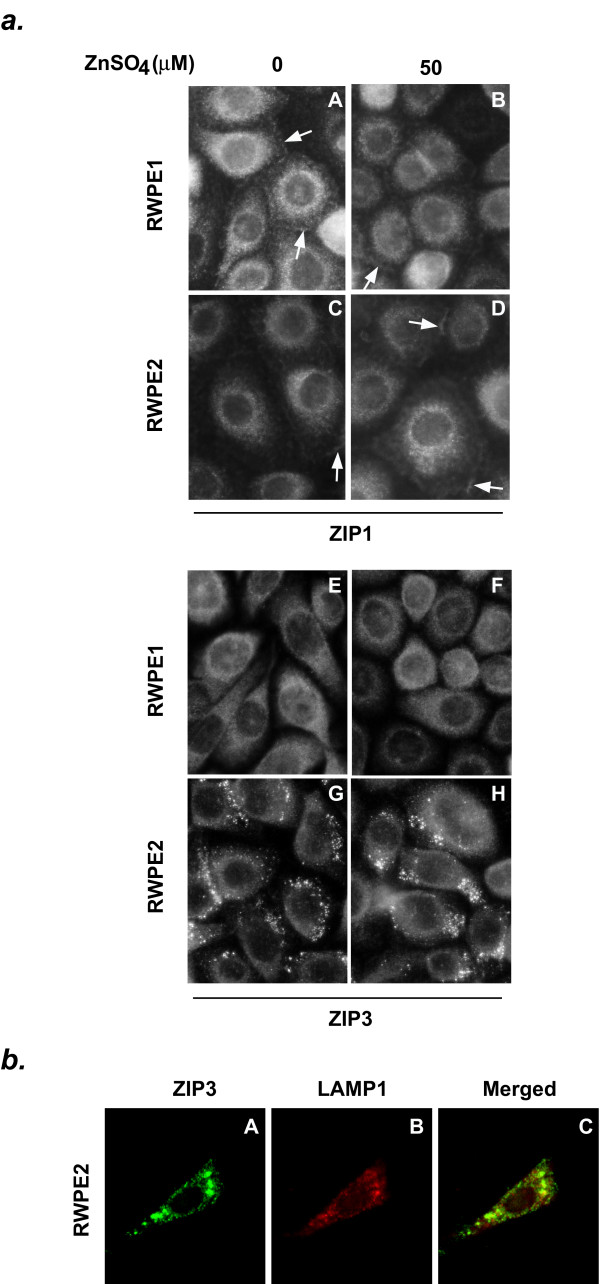
Subcellular localization of ZIP1 and ZIP3 in RWPE1 and RWPE2. *a*. Localization of  ZIP1 and ZIP3. RWPE1 and RWPE2 were seeded at ~50% confluence in a 4-well slide chamber and cultured at 37°C for 48 hours. Cells were then treated with 0 or 50 μM ZnSO_4 _at 37°C for 2 hours before immunofluorescent staining. The endogenous ZIP1 and ZIP3 proteins in RWPE1 and RWPE2 were detected by a chicken anti-ZIP1 antibody and a rabbit anti-ZIP3 antibody, respectively. An Alexa 488-conjugated goat anti-chicken (ZIP1) or anti-rabbit (ZIP3) antibody were used as secondary antibodies for the photomicrographs. Arrows indicate the plasma membrane localization of ZIP1. *b*. Localization of ZIP3 and LAMP1 in RWPE2. RWPE2 were seeded at ~50% confluence in a 4-well slide chamber and cultured at 37°C for 48 hours. Cells were fixed, permeablized, and stained as described in the Methods. The *green *(ZIP3, panel A) and *red *(LAMP1, panel B) florescent images were merged to indicate the area of overlap (panel C), which is shown in *yellow*.

The disrupted zinc homeostasis in RWPE2 may be caused by the changes in protein trafficking of zinc transporters. To test this hypothesis, expression and localization of ZIP1, ZIP3, ZNT1-2, and ZNT4-7 in RWPE1 and RWPE2 were examined by immunofluorescence microscopy. No detectable difference in protein distributions and intensities of ZNT1-2 and ZNT4-7 between RWPE1 and RWPE2 were observed (data not shown). The cellular distribution of ZIP1, which was localized on the cytoplasmic membrane as well as intracellular vesicles, is similar between RWPE1 and RWPE2 (Fig. 4a, panels A and C). Zinc treatment (50 μM ZnSO_4 _at 37°C for 2 hours) has little effect on the cellular localization of ZIP1 in RWPE1 (Fig. 4a, panel B) and RWPE2 (Fig. 4a, panel D). The ZIP3 protein was localized throughout RWPE1 (Fig. 4a, panel E). It was intriguing that numerous strong stained particles were detected in RWPE2 with the anti-ZIP3 antibody in addition to the vesicle staining observed in RWPE1 (Fig. 4a, panel G). The distribution pattern of these fluorescent particles resembles the localization of the lysosomal compartment [32]. To conform the localization of the punctate staining of ZIP3 in RWPE2, we compared the cellular localizations of ZIP3 and LAMP1, a lysosome-associated membrane protein [33], in RWPE2 by immunofluorescent co-localization assay. As shown in Fig. 4b, most punctate staining of ZIP3 (Fig. 4b, panel A) was overlapping with that of LAMP1 (Fig. 4b, Panel C) in RWPE2, strongly suggesting that the ZIP3 protein was compartmentalized in lysosomes in these cells. Furthermore, the cellular localization of ZIP3 was not affected by zinc treatment (50 μM ZnSO_4 _at 37°C for 2 hours) in RWPE1 and RWPE2 (Fig. 4a, panel F and H, respectively).

### Decrease in the protein expression of ZIP1 in RWPE2

**Figure 5:**
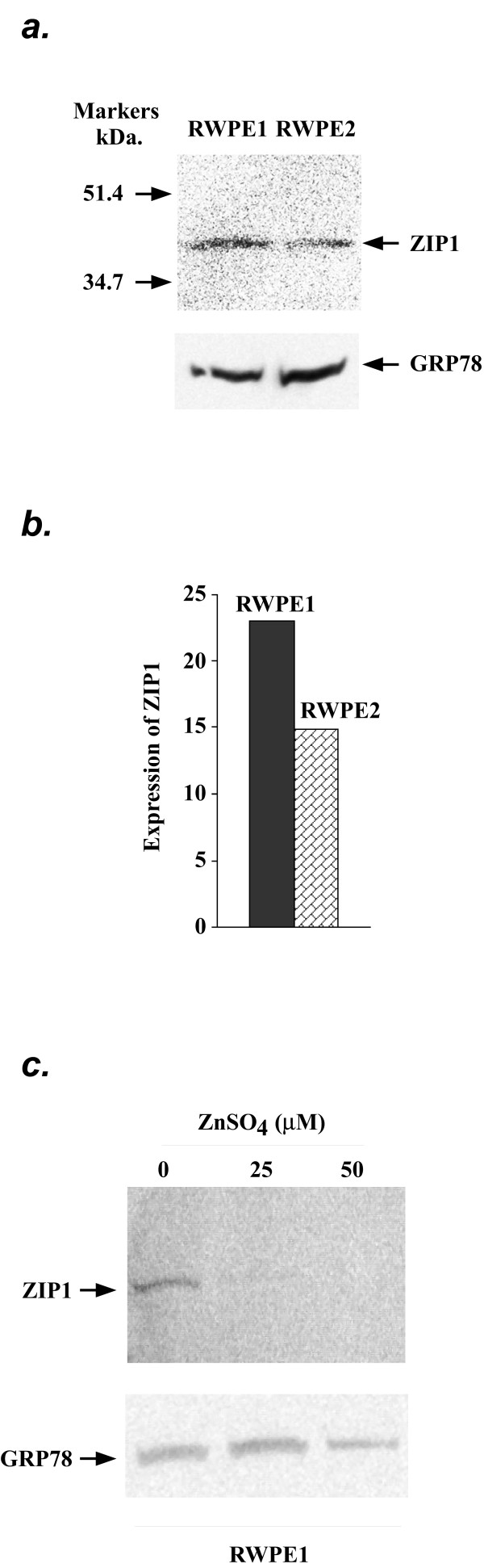
Expression of the ZIP1 protein in RWPE1 and RWPE2. *a*. Detection of the ZIP1 protein. Western blot containing 50 μg of protein extracts isolated from RWPE1 and RWPE2 cells were probed with a chicken anti-ZIP1 antibody followed by a peroxidase-conjugated secondary anti-chicken antibody. ZIP1 was visualized using an ECL kit (Amersham Biosciences). The detection of GRP78 on the same western blot was served as loading control. The protein markers (Bio-Rad) are shown. *b*. Quantitation of the expression level of the ZIP1 protein. The ZIP1 and GRP78 signals from the western blot (Fig. 5a) were quantified by an Alpha Innotech Gel Documentation System (Alpha Innotech). The expression of ZIP1 was normalized by the expression of GRP78. *c*. Regulation of the ZIP1 protein expression by zinc. RWPE1 cells were grown for 24 hours and treated with 0, 25, or 50 μM ZnSO_4 _for 24 hours. Cell lysate was prepared and analyzed by western blot assay. The ZIP1 and GRP78 proteins were detected as described as above.

It has been demonstrated lately that the ZIP1 protein expression was down-regulated in the malignant prostate tissues [34]. This prompted us to examine and compare the protein expression levels of ZIP1 as well as ZIP3 in RWPE1 and RWPE2 by western blot analyses. We found that the ZIP1 protein expression was down-regulated ~36% in RWPE2 compared to that in RWPE1 (Fig. 5a &5b) whereas the protein expression of ZIP3 was comparable between RWPE1 and RWPE2 (data not shown). Moreover, the expression of the ZIP1 protein was regulated by extracellular zinc. As shown in Fig. 5c, the levels of ZIP1 dramatically decreased in the RWPE1 cells treated with 25 μM or 50 μM ZnSO_4 _for 24 hours as compared to these treated with 0 μM ZnSO_4_. Zinc had no effect on the protein expression levels of ZIP3 in RWPE1 and RWPE2 (data not shown). Taken together, the results suggested that the regulatory mechanism of ZIP1 and ZIP3 are distinctive in prostate epithelial cells.

### Generation of RWPE2 cell lines carrying vector control or ZIP1-Myc plasmid

**Figure 6:**
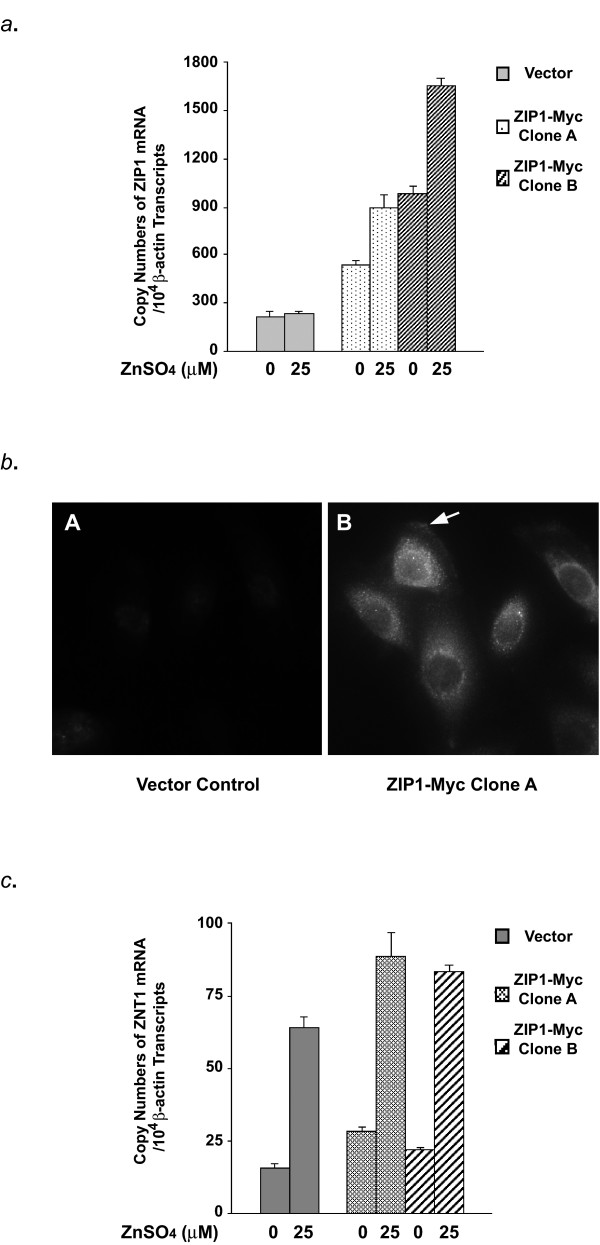
Over-expression of ZIP1-Myc in RWPE2. RWPE2 was stably transfected with an empty vector or a ZIP1-myc expressing plasmid. *a*. Expression of the ZIP1-Myc mRNA in two stably transfected RWPE2 cell lines. Vector or ZIP-Myc transfected (Clone A and Clone B) RWPE2 were seeded at ~50% confluence and 0 or 25 μM ZnSO_4 _was added into the medium after 24 hours incubation. Cells were then incubated at 37°C for another 24 hours and total RNA was isolated. The endogenous ZIP1 as well as ZIP1-Myc mRNA were measured by quantitative RT-PCR with a TaqMan probe specific for ZIP1. The expression of ZIP1 mRNA was normalized to the β-actin mRNA expression. Values are the means ± SD of three experiments. *b*. Expression and localization of ZIP1-Myc in stably transfected RWPE2. Vector and ZIP1-Myc transfected RWPE2 were seeded at ~25% confluence and cultured at 37°C for 48 hours. ZIP1-Myc was detected by a mouse anti-Myc antibody (panel B). Arrow indicates the plasma membrane localization of ZIP1-Myc. The Myc staining of RWPE2 stably transfected with the vector control is shown in panel A. *c*. Expression of ZNT1 mRNA in stably transfected RWPE2. Vector or ZIP1-Myc expressing (Clone A and Clone B) RWPE2 were seeded at ~50% confluence and 0 or 25 μM ZnSO_4 _was added into the medium after 24 hours incubation. Cells were then incubated at 37°C for another 24 hours and total RNA was isolated. The endogenous ZNT1 mRNA was measured by quantitative RT-PCR with a TaqMan probe specific for ZNT1. The expression of ZNT1 was normalized to the β-actin expression. Values are the means ± SD of three experiments.

Our studies in ^65^zinc uptake and immunofluorescent assays suggested that the low accumulation of intracellular zinc in RWPE2 may partially be due to the down-regulation of zinc uptake transporter protein expression (Fig. 5). We sought to over-express the ZIP1 protein in RWPE2 to examine the effects of elevated intracellular zinc levels on cell proliferation and apoptosis. RWPE2 stably transfected with a vector or a ZIP1-Myc expressing plasmid, in which the Myc epitope was tagged at the C-terminal end of the human ZIP1 protein, were established. The levels of the endogenous ZIP1 as well as the ZIP-Myc transcripts were examined by quantitative RT-PCR with a TaqMan probe specific for ZIP1 (Applied Biosystems). As shown in Fig. 6a, the expression of ZIP1 mRNA was 2.5- and 4.5-fold higher in the ZIP1-Myc expressing RWPE2 Clone A and Clone B, respectively, than that in the control RWPE2. Zinc treatment (25 μM ZnSO_4 _for 24 hours) led to a further increase (~2-fold) of ZIP1-Myc expression in the ZIP1-Myc expressing RWPE2 cells due to the stimulation of the CMV promoter which controls the transcription of the ZIP1-Myc gene by zinc (Fig. 6a and [25]). An immunofluorescent staining assay using a mouse anti-Myc antibody indicated that the ZIP1-Myc fusion protein was expressed in the ZIP1-Myc stably transfected RWPE2 cells (Fig. 6b). The staining results also indicated that the ZIP1-Myc fusion protein was distributed normally as the endogenous ZIP1 protein (Fig. 4a, panel A).

It has been demonstrated that the expression of ZNT1 mRNA is up-regulated by elevated intracellular zinc concentrations [29,35]. Thus, we used ZNT1 mRNA expression levels to confirm that over-expression of ZIP1-Myc in RWPE2 resulted in increased cellular zinc levels in these cells. The ZNT1 mRNA was measured by quantitative RT-PCR with a TaqMan probe specific for ZNT1 (Applied Biosystems). As shown in Figure 6c, the ZNT1 expression levels in the ZIP1-Myc expressing RWPE2 were about 1.8-fold (Clone A) and 1.4-fold (Clone B) higher than that in the vector transfected RWPE2, respectively, under the normal culture conditions. In addition, the expression of ZNT1 in both vector and ZIP1-Myc transfected RWPE2 was up-regulated by zinc (Fig. 6c). Taken together, these results suggest that over-expression of the ZIP1-Myc protein in RWPE2 results in an increase of intracellular labile zinc level which was sensed by the ZNT1 gene.

### Inhibition of cell growth by over-expression of ZIP1 in RWPE2

The mortality of prostate cancer is highly associated with tumor cell proliferation and apoptosis. The faster cell proliferation and/or lesser apoptosis the tumor is, the higher mortality it is. Therefore, the pathways involved in cell proliferation and apoptosis have been focused on for potential intervention of prostate cancer progression. In order to determine the effects of increased intracellular zinc in the ZIP1-Myc expressing RWPE2 on cell growth, we performed an *in vitro *cell growth assay with or without zinc (0 or 25 μM ZnSO_4_) added into the culture medium. We used 0 μM ZnSO_4 _treatment to represent a normal culture condition and 25 μM zinc to mimic the zinc supplemental condition. The normal human serum zinc values are about 9–16 μM [36].

**Figure 7:**
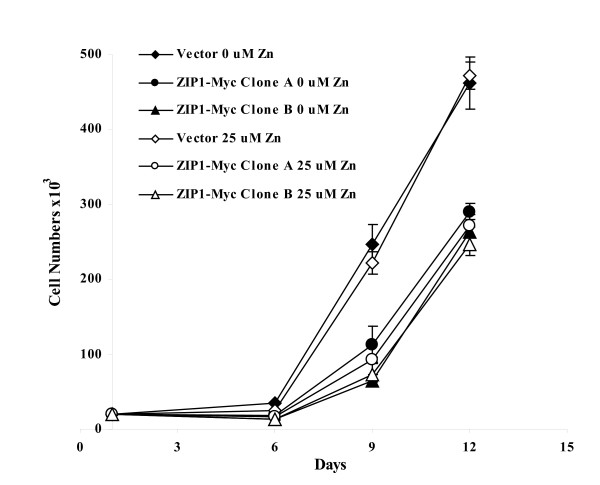
Effect of over-expression of ZIP1-Myc on RWPE2 cell growth. Vector or ZIP1-Myc expressing (Clone A and Clone B) RWPE2 were plated at a density of 20,000 per well in 12-well plates for 24 hours and then 0 or 25 μM ZnSO_4 _was added into the medium. Medium was changed at days 3, 6 and 9 after plating. Cells were harvested and cell numbers were determined at days 6, 9, and 12 after plating. Values are the means ± SD of three experiments.

The growth of the ZIP1-Myc expressing RWPE2 and their vector control were monitored for 12 days at 37°C in the presence of 0 or 25 μM ZnSO_4 _in the culture medium. The culture medium was changed at days 3, 6, and 9 after cells were seeded. Cells were harvested and cell numbers were determined at days 6, 9, and 12 after plating. As shown in Fig. 7, the growth of the ZIP1-Myc expressing RWPE2 was 37% (Clone A) and 43% (Clone B) slower than their vector control (*p *< 0.01). Zinc treatment (25 μM ZnSO_4_) did not further inhibit the growth of the ZIP1-Myc expressing RWPE2. In addition, there was no effect of extracellular zinc (25 μM ZnSO_4_) on the growth of the vector control.

### Increased apoptosis in RWPE2 over-expressing ZIP1

**Figure 8:**
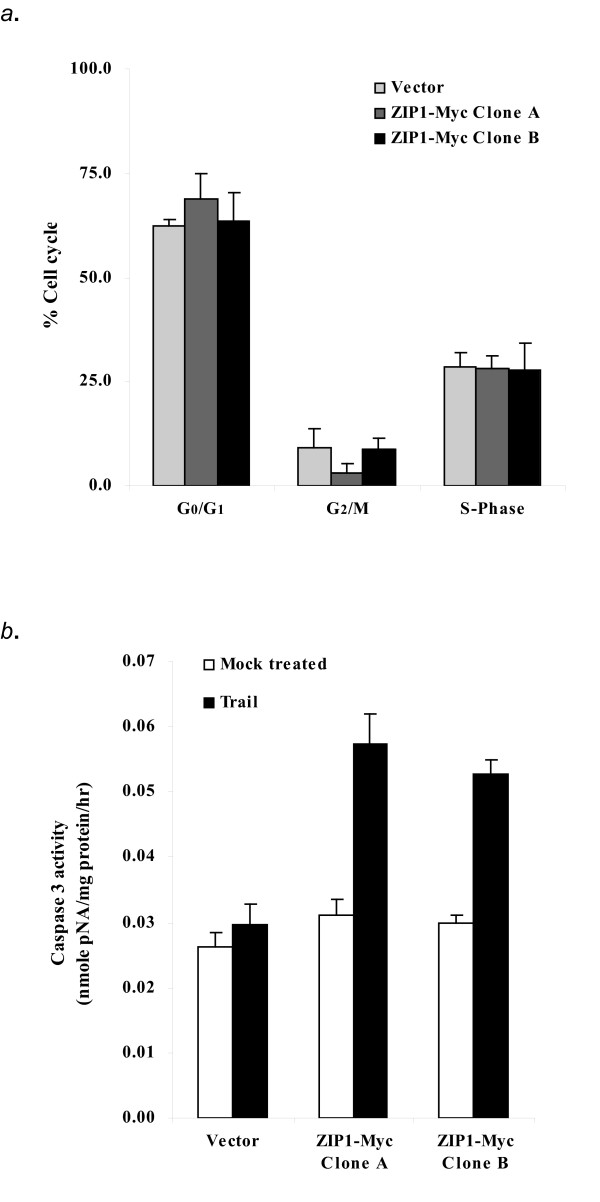
Effect of over-expression of ZIP1-Myc on RWPE2 cell cycle progression and apoptosis. *a*. Effect of over-expression of ZIP1 on cell cycle. Vector or ZIP1-Myc expressing RWPE2 cells were plated and grown for 24 hours. Cells were harvested and counted. 2 × 10^6 ^cells were ethanol fixed and processed for DNA staining with propidium Iodine. Cells were run on a FACS SCAN (BD Dickenson) with a 488 nm argon laser light source. 10,000 events were counted per sample. Values are the means ± SD of one representative experiment of two with similar results. *b*. Effect of over-expression of ZIP1 on apoptosis. 2 × 10^6 ^vector or ZIP1-Myc expressing RWPE2 cells were plated in 100 mm plates and grown for 24 hours. Cells were treated with TRAIL (10 ng/ml) at 37°C for 6 hours. Cells were harvested by trypsinization. Caspase 3 activity was determined according to the manufacture's instructions (Clontech ApoAlert Caspase Colorimetric Assay Kit). Values are means ± SD of one representative experiment of two with similar results.

To address pathways that led to slower growth of ZIP1 over-expressing RWPE2, we examined the cell proliferation rate by cell cycle progression and programmed cell death by caspase 3 activity. We found that over-expression of ZIP1 in RWPE2 did not affect cell cycle progression of RWPE2 (Fig 8a). Although the mean percentage of G_2_/M of RWPE2 over-expressing ZIP1 (clone A) was lower than that of the vector control, it was not statistically significant (*p *> 0.05). We then examined the apoptotic death of vector or ZIP1 over-expressing RWPE2 by analyzing caspase 3 enzymatic activity. Caspase 3 is one of the intracellular cysteine proteases that are synthesized as zymogens and then proteolytically activated early in the course of apoptosis [37]. Vector control and ZIP1 over-expressing RWPE2 were treated with TRAIL (tumor necrosis factor a-related apoptosis-inducing ligand) at 37°C for 6 hours and the caspase 3 activities were measured as described in the Methods. TRAIL is an apoptotic inducing protein that binds to its receptors (DR4 and DR5) to transduce an apoptotic signal to the downstream apoptotic pathway. After 6 hours TRAIL treatment, control RWPE2 cells were resistant to the low concentration of TRAIL (10 ng/ml, Fig. 8b). This result is consistent with the observation [38] that human prostate cancer cell lines including PC3, DU145, and LNCaP were not sensitive to the TRAIL treatment. However, over-expression of ZIP1 in RWPE2 cells made cells (Clone A & B) sensitive to the TRAIL-induced apoptosis (~90% increase, Fig. 8b), suggesting that an increase of intracellular zinc concentration via zinc uptake protein in malignant prostate epithelial cells may be useful for the treatment of prostate cancer.

## Discussion

The uniqueness of the normal mammalian prostate epithelial cells is their ability to accumulate high cellular zinc. However, malignant prostate epithelial cells lose this function [8,9,34]. To better understand the role of zinc in the development and progression of prostate cancer, we investigated and compared the features of a non-tumorigenic and a tumorigenic human prostate epithelial cell lines in aspects of zinc accumulation, zinc uptake, zinc transporter expression profiles, and functional consequences of over-expression of a major prostate zinc uptake protein, ZIP1. In this study, we used two existing cellular models, the non-tumorigenic human prostate epithelial cells, RWPE1, and its tumorigenic counterpart, RWPE2, because both cell lines essentially share the same genetic background. We demonstrated that both RWPE1 and RWPE2 possess the ability to accumulate zinc when zinc was added into the culture medium. However, the levels of the accumulated zinc in RWPE2 were 39% and 48% lower than that of RWPE1 in a total cell-associated zinc assay and a ^65^Zn accumulation assay, respectively. These findings confirmed the clinic observation that the prostate cancer cells contained less cellular zinc than the normal epithelial cells in the surrounding areas, indicating that the function of zinc accumulation in malignant prostate epithelial cells is impaired during tumorigensis [1,8,9,34]. Our results also demonstrated that RWPE1 and RWPE2 are excellent *in vitro *cellular models for studying intracellular zinc homeostasis of prostate epithelial cells under the normal and malignant conditions.

The tight homeostatic control of cellular zinc is largely accomplished by the regulation of zinc influx and efflux systems. Thus, less zinc accumulation in malignant prostate epithelial cells may result from reduced zinc uptake and/or increased zinc export. In this study, we conclude that the zinc uptake system rather than the zinc efflux system of RWPE2 is impaired during tumorigensis. This conclusion is based on several independent observations. First, we found that the initial rate of zinc uptake of RWPE2 was decreased 33% compared to its non-tumorigenic counterpart (Fig. 2). The level of the reduction in zinc uptake of RWPE2 is in agreement with that of the reduction in total cell-associated zinc accumulation. Second, the protein expression level of ZIP1, a major zinc uptake protein in prostate epithelial cells [39], was down-regulated in RWPE2 (Fig. 5). This result is consistent with the observation that the ZIP1 protein was down-regulated and cellular zinc was depleted during prostate cancer progression in the clinical specimens of human prostate cancer tissues [34]. Third, perhaps the significant discovery is that the ZIP3 protein may be subjected to degradation in RWPE2 as ZIP3 was detected in a lysosomal compartment (Fig. 4). Finally, we found no evidence showing that the regulations of transcription, translation, and protein trafficking of the ZNT genes including ZNT1-2 and ZNT4-7 were disrupted in RWPE2 (data not shown).

ZIP1 and ZIP3 have been demonstrated to be involved in translocation of extracellular zinc into a variety of cell types [24,26,30,39,40]. Inhibition of the activities of ZIP1 and ZIP3 leads to reduction of zinc uptake [24,26,39,40]. Our study suggests that the inhibitory mechanisms for ZIP1 and ZIP3 transport activities may not be the same in prostate epithelial cells. We demonstrate that the regulatory mechanism for the ZIP1 activity is exerted at the translational level in prostate epithelial cells. Higher cellular zinc concentrations or tumorigenic state of prostate epithelial cells decreased protein synthesis of ZIP1 whereas no effect was detected on the expression of mRNA and the cellular localization of ZIP1. On the other hand, the activity of ZIP3 was mainly regulated by the tumorigenic state of prostate epithelial cells. In the tumorigenic prostate epithelial cells (RWPE2), ZIP3 was compartmentalized to the lysosome, where we speculate ZIP3 is subjected to degradation. In addition, zinc has no effects on the mRNA and protein expression and cellular localization of ZIP3 in prostate epithelial cells. The domains mediating the regulations of protein expression and protein trafficking of ZIP1 and ZIP3 are not clear. Further studies are needed to determine these domains in ZIP1 and ZIP3 and their regulatory mechanisms.

Reduction in the protein expression of ZIP1 observed in RWPE2 led us to investigate the effects of over-expression of ZIP1 in RWPE2 on cellular zinc accumulation, cell growth, cell proliferation, and apoptosis. We demonstrate that over-expression of ZIP1 in RWPE2 increased intracellular zinc concentrations and inhibited cell growth in both zinc-adequate and excess conditions. We also show that the slower cell growth of ZIP1 over-expressing RWPE2 resulted from increased apoptosis in these cells. The mechanism(s) underlying the enhanced sensitivity of ZIP1-Myc over-expressing RWPE2 to TRAIL which led to the activation of caspase 3 have yet to be identified. A wide-range of apoptotic associated proteins which have been demonstrated to play important roles in regulation of TRAIL-mediated apoptosis are the primary targets for the future study. These include 1) the death receptors (DR4 and DR5) and the decoy receptors (DcR1, DcR2, and OPG) expressed on the surface of ZIP1-Myc over-expressing RWPE2; 2) the expression of NF-kappaB, Bax/Bak, Bcl-2/Bcl-xL, and p53 in ZIP1-Myc over-expressing RWPE2.

## Conclusion

RWPE1 and RWPE2 cell lines are excellent cellular models to study the association of intracellular zinc levels with prostate cancer progression *in vitro*. Tumorigenic prostate epithelial cells (RWPE2) accumulate less intracellular zinc compared to the non-tumorigenic counterpart. The reduction in capacity for accumulation of cellular zinc in tumorigenic prostate epithelial cells may be caused by the decrease of ZIP1 expression and the redistribution of ZIP3. The observation that increasing cellular zinc level by over-expression of the zinc uptake protein, ZIP1, inhibits tumorigenic prostate epithelial cell growth through apoptosis may provide a basis for a potential therapeutic strategy for prostate cancer.

## Methods

### Cell culture and generation of stable cell lines

Human prostate epithelial cell lines (RWPE1 and RWPE2) were purchased from American Tissue Collection Center (ATCC) and were maintained in the Keratinocyte-serum free medium (K-SFM) containing 5 ng/ml human recombinant epithelial growth factor (hrEGF), 0.05 mg/ml bovine pituitary extract (Invitrogen), 100 U/ml Penicillin G, and 100 μg/ml Streptomycin. The zinc contents for the Keratinocyte-Serum Free medium with supplements were about 0.7 μM determined by a Vista AX Simultaneous ICP-AES (Varian). Both RWPE1 and RWPE2 grew adherently in tissue culture. The ZIP1-Myc expressing cell line and vector control cell line were generated by transfecting pcDNA3.1/ZIP1-Myc or pcDNA3.1/Myc-His (Invitrogen) into RWPE2 using LipofectAMINE plus kit (Invitrogen). The stable cell lines were selected by culturing the cells in the medium containing 400 μg/ml of G418 (Invitrogen).

### Plasmid construction

The human EST clone (BI820953) containing the *ZIP1 *cDNA sequence was purchased from ATCC. The *ZIP1 *open reading frame (ORF) sequence lacking a stop codon was amplified using a forward primer with a *Hind*III site incorporated 11 base pairs upstream of the methionine codon (5'-TCACTGAAGCTTCCAGAAGCATC-3') and a reverse primer with a *Xho*I site incorporated in the stop codon (5'-GAAGCCCTCGAGATTTGGATG-3'). The PCR product was purified, digested with *Hind*III and *Xho*I restriction enzymes, and inserted into the respective enzyme sites of a mammalian expression vector, pcDNA3.1/Myc/His, version B (Invitrogen). The resulting plasmid pcDNA3.1/ZIP1-Myc was sequenced and used for generation of a stable RWPE2 cell line expressing ZIP1-Myc.

### Measurement of total cell-associated zinc contents

RWPE1 and RWPE2 were seeded into 100-mm culture plates at 2 × 10^6 ^cells per plate. Cells were treated with 0 or 75 μM ZnSO_4 _at 37°C for 48 hours after cells reached to ~50% confluence. Cells were then harvested and washed four times with 1× PBS, pH 7.4. Cell numbers were determined by measuring the absorbance of cell suspensions at OD600 nm (A_600_) and converting to cell number based on a standard curve. The total cell-associated zinc in RWPE1 and RWPE2 was then measured by a Vista AX Simultaneous ICP-AES (Varian) using nitric acid digestion method [10].

### Zn uptake and accumulation assays

RWPE1 and RWPE2 were grown in T-150 flasks for 72 hours (~70% confluence). Cells were treated with 1× trypsin (Invitrogen) and neutralized with DMEM containing 10% FBS. Cells were then harvested by centrifugation and washed once in cold 1× PBS, pH 7.4, and once in cold uptake buffer (15 mM HEPES, 100 mM glucose, 150 mM KCl, pH 7.0) [25]. Cells were resuspended in the uptake buffer and cell numbers were determined by the standard curve method. Tubes containing 3 × 10^5 ^cells per tube (150 μl in total volume) were placed in a shaking incubator and incubated at 37°C for 10 minutes. Then an equal volume of pre-warmed uptake buffer containing 15 pmol ^65^ZnCl_2_/150 μl (Oak Ridge National Laboratory, Oak Ridge, TN) was added and incubated for the indicated time points. Reactions were stopped by adding an equal volume of ice-cold stop buffer (15 mM HEPES, 100 mM glucose, 150 mM KCl, 1 mM EDTA, pH 7.0) [25]. Cells were collected by filtration on glass microfiber filters (GF/C, Whatman) and washed three times with cold stop buffer (12 ml total wash volume). Cell-associated radioactivity was determined with a gamma counter.

### Total RNA isolation and cDNA synthesis

RWPE1, RWPE2, and RWPE2/ZIP1-Myc were grown for 24 hours and then treated with 0 or 75 μM ZnSO_4 _for 48 hours before harvesting. The total RNA was purified by a micro total RNA purification kit (Invitrogen). The cDNA was synthesized from 3 μg total RNA using a SuperScript Choice system (Invitrogen).

### Quantitative RT-PCR analysis

cDNA was diluted 4-fold and 2 μL cDNA was added to a quantitative PCR mixture containing corresponding primer pair and a FAM-labeled TaqMan probe (Applied Biosystems). The quantitative PCR reactions were performed on a PRISM^® ^ABI 7900HT Sequence Detection System (Applied Biosystems) in triplicate and the expression of β-actin (ACTB) was used for normalization. Copy numbers for the zinc transporter genes were calculated using a standard curve method and normalized to the copy numbers of ACTB.

### Generation of standard curves

The amplicons generated with Assays-on-Demand primer sets for ACTB, ZIP1, ZIP3, and ZNT1 (Applied Biosystems) were inserted into the cloning vector pCR2.1-TOPO following the manufacturer's protocol (Invitrogen). The identity of all plasmids was confirmed by sequencing. Plasmids were then linearized by either *EcoRI *or *XhoI *restriction enzyme digestion before making a series of ten-fold dilutions (10^3 ^to 10^8^) for establishing standard curves for mRNA quantification.

### Antibodies

A chicken anti-ZIP1 and a rabbit anti-ZIP3 antibodies were kindly given by Dr. Shannon Kelleher (Department of Nutrition, University of California at Davis) [40]. A mouse anti-GRP78 was obtained from Transduction Laboratories. Mouse anti-Myc and anit-LAMP1 monoclonal antibodies were purchased from StressGen. Alexa 488-conjugated goat anti-chicken, anti-rabbit, and anti-mouse antibodies were purchased from Molecular Probes. Peroxidase-conjugated goat anti-mouse, anti-chicken, and anti-rabbit antibodies were purchased from PIERCE.

### Immunofluorescence microscopy

Immunofluorescence analysis was performed as described [22]. RWPE1, RWPE2, and RWPE2 expressing vector control or the ZIP1-Myc fusion protein were cultured in slide chambers for 48 hours, fixed with 4% paraformaldehyde, and permeablized with 0.4% saponin (Sigma). RWPE1 and RWPE2 cells were stained with an anti-ZIP1 or an anti-ZIP3 antibody (1:100 dilution) followed by an Alexa 488-conjugated goat anti-chicken or anti-rabbit antibody (1:500 dilution). In the co-localization assay, RWPE2 were stained anti-ZIP3 (1:100 dilution) and anti-LAMP1 (1:50 dilution) antibodies followed by Alexa 488-conjugated goat anti-rabbit (1:500 dilution) and Alexa 594-conjugaged goat anti-mouse antibodies (1:500 dilution). RWPE2 cells expressing vector or ZIP1-Myc were stained with an anti-Myc antibody (1:250 dilution) followed by an Alexa 488-conjugated goat anti-mouse antibody (1:500 dilution). Photomicrographs were obtained by a Nikon Eclipse 800 microscope with a digital camera.

### Western blot analysis

RWPE1 and RWPE2 cells were cultured in a Keratinocyte-Serum Free medium supplemented with 5 ng/ml human recombinant EGF and 0.05 mg/ml bovine pituitary extract (Invitrogen) at 37°C for 48 hours. For zinc treatment, RWPE1 cells were cultured in the medium described above at 37°C for 24 hours. The cells were then cultured in the medium containing 0, 25, or 50 μM ZnSO_4 _for another 24 hours at 37°C. Cells were harvested and cell lysate was prepared as previously described [22]. 50 μg protein extracts were separated on a 4–20% Tris-HCl gel (Bio-Rad) and transferred to a nitrocellulose membrane (Bio-Rad). The ZIP1 and GRP78 proteins were detected by a chicken anti-ZIP1 antibody (1:5,000 dilution) and a mouse anti-GRP78 (1:6,000 dilution), respectively, followed by a peroxidase-conjugated secondary antibody (1:2,500-10,000 dilution) (PIERCE). ZIP1 and GRP78 were visualized by using an ECL kit (Amersham Biosciences) and an Alpha Innotech Gel Documentation System (Alpha Innotech).

### Cell growth

RWPE2/ZIP1-Myc and RWPE2/vector cells were plated in 12-well plates at a density of 20,000 cells/well in triplicate. Zinc treatment (25 μM ZnSO_4_) started at day 2 after plating. Medium was changed every 3 days. Cells were washed twice with the culture medium to remove dead cells and harvested by typsinization at days 6, 9, and 12 after plating. The cell suspension homogeneity was confirmed under microscope after trypsinization and resuspension. In addition, the percentages of viable cells in these suspensions were assessed by trypan blue exclusion. Finally, cell numbers were determined by measuring the cell suspensions at OD_600 _using a standard curve for RWPE2. The standard curve for RWPE2 cells was established by counting cells using a hemacytometer and measuring the absorptions at OD600 after typsinization and resuspension.

### Cell cycle analysis

Vector or ZIP1-Myc expressing RWPE2 were plated and grown for 24 hours. Cells were then harvested and counted. 2 × 10^6 ^cells were ethanol fixed and processed for DNA staining with propidium Iodine as described [41]. Cells were run on a FACS SCAN (BD Dickenson) with a 488 nm argon laser light source. 10,000 events were counted per sample.

### Caspase 3 activity analysis

2 × 10^6 ^vector or ZIP1-Myc expressing RWPE2 were plated in 100 mm plates and grown for 24 hours. Cells were then treated with TRAIL (10 ng/ml) at 37°C for 6 hours. After treatment, cells were harvested by trypsinization and suspended cells were collected by centrifugation. Cells were then processed for Caspase 3 activity assay using an ApoAlert Caspase Colorimetric Assay Kit (Clontech). Caspase 3 activity was determined colorimetricly at 405 nm according to the manufacture's instructions.

### Statistical analysis

The significance of differences between sample groups was calculated using a paired Student's *t*-test with a two-tailed distribution. Differences were considered significant at *p *< 0.05. Data are means ± SE or SD.

## Abbreviations

ZnT = zinc transporter, ZIP = ZRT, IRT-like protein family, EST = expressed sequence tag, CDF = cation diffusion facilitator, TRAIL = tumor necrosis factor a-related apoptosis-inducing ligand.

## Competing interests

The author(s) declare that they have no competing interests.

## Authors' contributions

LH carried out measuring cellular zinc contents, uptake, and accumulation, establishing stable cell lines, and monitoring cell growth rates. LH also drafted and finalized manuscript. CPK carried out plasmid constructions, part of total RNA isolation and cDNA synthesis experiments, immunofluorescence microscopy assay, western blot analysis, cell cycle analysis, and caspase 3 activity analysis. YZ performed part of total RNA isolation and cDNA synthesis experiments, generation of standard curves for RNA quantification, and quantitative RT-PCR analysis.
